# Barriers and facilitators to the implementation of integrated disease surveillance and response (IDSR) in Africa: a systematic review using the consolidated framework for implementation research (CFIR)

**DOI:** 10.3389/fpubh.2026.1758944

**Published:** 2026-02-20

**Authors:** Dzinkambani Moffat Kambalame, Trude Margrete Arnesen, Jim Mtambo, Evelyn Chitsa Banda, John Phuka, Adamson Sinjani Muula

**Affiliations:** 1 Department of Community and Environmental Health, School of Global and Public Health, Kamuzu University of Health Sciences, Blantyre, Malawi; 2 Public Health Institute of Malawi (PHIM), Ministry of Health, Lilongwe, Malawi; 3 Norwegian Institute of Public Health (NIPH), Oslo, Norway; 4 Library Department, Kamuzu University of Health Sciences, Lilongwe, Malawi

**Keywords:** Africa, CFIR, IDSR, implementation research, integrated disease surveillance, systematic review

## Abstract

**Background:**

Integrated Disease Surveillance and Response (IDSR) are crucial for strengthening public health systems in Africa but faces implementation challenges. Despite the growing utility of implementation theories, models, and frameworks in assessing the determinants of innovation implementation, no implementation research theories have examined the determinants of IDSR implementation. This systematic review aimed to identify the barriers and facilitators of IDSR implementation in Africa using a Consolidated Framework for Implementation Research (CFIR).

**Methods:**

We systematically searched four databases, EMBASE, PUBMED, CINAHL, and Scopus, for studies on IDSR assessments in Africa published between 2010 and 2025. Data on barriers and facilitators were extracted and mapped to CFIR domains and constructs and analyzed deductively using content analysis and inductively using thematic analysis. The quality of the included studies was assessed using the Johns Hopkins Nursing Evidence Appraisal Tool. The review was reported in alignment with Preferred Reporting Items for Systematic Reviews and Meta-Analysis (PRISMA).

**Results:**

Thirty-seven studies were included in this analysis. Six CFIR domains and 24 constructs explained the determinants of IDSR implementation. The key barriers identified were limited resources, inadequate access to knowledge/information, and structural constraints in the inner-setting domain. Individual domain factors, such as low motivation and capability, also emerged as crucial barriers. Facilitators included structural characteristics, improved access to knowledge, and adequate resources in the inner setting domain as well as positive innovation outcomes in the outcome domain.

**Conclusion:**

The implementation of IDSR encounters complex barriers and opportunities within health facilities. Comprehensive strategies addressing both organizational and individual factors of healthcare facilities are needed. The CFIR framework, alongside the CFIR-ERIC matching tool, provides a foundation for identifying context-specific implementation strategies that lead to improved innovation performance.

**Systematic Review Registration:**

PROSPERO https://www.crd.york.ac.uk/PROSPERO/view/CRD42024571576, identifier CRD42024571576.

## 1 Introduction

Integrated Disease Surveillance and Response (IDSR) are vital for strengthening national public health systems and enabling timely responses to disease outbreaks. It was established in 1998 by the World Health Organization (WHO) Regional Committee for Africa (1) following recurrent outbreaks of cholera, yellow fever, and dengue in the 1990s (1). The purpose was to provide a framework for establishing an effective national public health surveillance system for countries in the WHO/AFRO region. The key features of IDSR include the integration of disease surveillance, application of standard guidelines, and the analysis and use of data for decision-making (2).

Despite its critical importance, the implementation of IDSR in Africa faces numerous systemic challenges (3, 4). Research has identified these challenges as pervasive across the continent, although there is variation in performance among countries. Some countries exhibit strong performance, whereas others continue to encounter difficulties (5). These studies have predominantly focused on evaluating the core and support functions as well as the attributes of the IDSR system, employing the World Health Organization (WHO) (6) and Centers for Disease Control and Prevention (CDC) (7) protocols for evaluating surveillance systems.

Despite the availability of research on the barriers and facilitators of IDSR implementation, the application of implementation research theories, models, and frameworks (8) to structure and analyze the determinants of IDSR implementation has been limited. Employing well-established implementation research frameworks such as the Consolidated Framework for Implementation Research (CFIR) ensures that the assessment of implementation determinants is informed and grounded in a robust theoretical foundation, thereby enhancing the credibility of findings (8). Furthermore, utilizing CFIR offers the advantage of applying tools such as the CFIR-ERIC matching tool to select strategies to improve the implementation of an intervention (9). Given this gap, there is a need to conduct a systematic review through the CFIR lens to enhance the understanding of barriers.

This systematic review aimed to identify the barriers and facilitators associated with the effective implementation of IDSR in African countries by employing the CFIR as a guiding analytical tool. By examining the obstacles to IDSR implementation through the CFIR framework, this study aimed to establish a foundation for selecting context-specific strategies to enhance the performance of the IDSR system in Africa and comparable settings. Our findings are intended to assist surveillance implementers in refining practices and aid health authorities in informing policy decisions.

## 2 Methods

### 2.1 Eligibility criteria

#### 2.1.1 Type of studies to be included

This review included studies that applied qualitative, quantitative, and mixed methods to assess barriers and facilitators to the implementation of the IDSR system from the perspective of health workers in Africa. The inclusion criteria were as follows: (1) primary, peer-reviewed, published, or gray literature research evaluating the IDSR system following its alignment with the International Health Regulations (IHR) 2005. Therefore, studies conducted in 2010 onward were included, and the review process was completed on June 30, 2025. (2) The setting is Africa. (3) The intervention of interest was a public health surveillance system guided by the IDSR, as described by the World Health Organization (WHO). The exclusion criteria were as follows: (1) studies that did not report any determinants (barriers or enablers) of IDSR system implementation. (2) Studies not published in English. The review protocol was registered in PROSPERO, with registration number CRD42024571576.

#### 2.1.2 Participants

Our review focuses on the perspectives of African health workers.

#### 2.1.3 Types of outcome measures

The primary objective of this systematic review was to identify the barriers associated with the implementation of the IDSR system from the perspective of health workers in Africa. This outcome was based on the findings of studies that evaluated IDSR implementation in African countries that adopted the IDSR guidelines from 2010 or later. The secondary outcomes of this review were facilitators or enablers for the implementation of IDSR systems.

#### 2.1.4 Intervention of interest

We focused on public health surveillance systems that had been developed based on the IDSR strategy. The IDSR system was launched in Africa as a strategy to strengthen national public health surveillance to effectively detect and respond to health threats (10). Following the revision of the International Health Regulations (IHR) in 2005 by the World Health Assembly, the IDSR guidelines were revised in 2010 to support the implementation of IHR in Africa. Therefore, the core and support functions of the IDSR system, guided by the second (11) and third editions of the IDSR guidelines (12), ensured alignment with the IHR, 2005 (13), which is a legally binding framework aimed at coordinating efforts to prevent, detect, and respond to health threats (14). The key activities of the IDSR system are organized into eight core functions: (1) identification, (2) reporting, (3) analysis and interpretation, (4) investigation and confirmation, (5) preparation, (6) responding, (7) communicating risk, and (8) monitoring, evaluating, supervising, and providing feedback for improvement (12).

### 2.2 Search method for identification of studies

We searched PubMed, Web of Science, Scopus, and the Cumulative Index for Nursing and Allied Health Literature (CINAHL). We manually screened the reference lists of the identified articles to identify additional studies.

We used a SPIDER framework to create a search strategy from the research question “*What barriers and facilitators influence the implementation of the IDSR system in Africa*?” We employed a combination of keywords and Medical Subject Headings (MeSH) pertinent to the phenomenon of interest (PI from the SPIDER framework), specifically focusing on the IDSR system in Africa. In addition, keywords and MeSH related to the evaluation (from the SPIDER framework) were incorporated.

In brief, we used the following keywords and MesH terms in different combinations: (“Integrated Disease Surveillance and Response,” “Integrated Disease Surveillance and Response System,” IDSR, “integrated disease surveillance,” “IDS,” “disease surveillance,” “surveillance,” “public health surveillance” [MeSH], “notifiable disease surveillance systems” “communicable disease surveillance systems”) AND (barriers, challenges, factors, facilitators, determinants) AND (“Africa”[MeSH] OR “Africa, Sub-Saharan”[MeSH]). Additional information regarding the search strategy is provided in Supplementary File 1.

### 2.3 Selection of studies

Two reviewers applied the eligibility criteria and selected the studies for inclusion in the systematic review. Initially, two reviewers independently screened the titles and abstracts of the retrieved studies for relevance to the main aim of the systematic review. Inclusion criteria were used to ensure that the selected studies met the required standards. We then retrieved the full text of potentially eligible studies for further screening to confirm that they met the eligibility criteria. The screening and selection of studies were conducted independently by two reviewers, and any disagreements were resolved by consensus. If a consensus was not reached, disputes were resolved by the senior author ASM. The Rayyan software was used to complete the task.

### 2.4 Data extraction and management

We extracted data from the included studies using a form developed in line with the Preferred Reporting Items for Systematic Reviews and Meta-Analyses (PRISMA) checklist (15). Data extraction was conducted independently by two reviewers using a data collection form adapted from a form used elsewhere (16). We extracted data on the following variables: author's name, year of publication, country where the study took place, study aim(s), study type, framework applied, sample description, and identified barriers and facilitators (Table 1). Data on the level of strength and quality of evidence were also extracted (see Supplementary File 2). Disagreements were resolved by consensus. If a consensus was not reached, disputes were resolved by the senior author ASM. Microsoft Excel was used to complete this task.

**Table 1A:** Study characteristics.

**Author (Year)**	**Country**	**Study design**	**Sample description**
Meierkord et al. (77)	Côte d'Ivoire, Ecuador, Madagascar, Namibia, and the Kingdom of Saudi Arabia	Qualitative; literature review, in-depth interview.	Sample size: 57 participants: professionals from government, NPHIs, academic institutions and the private sector. Interviews were thematically analyzed.
Rumunu et al. (78)	South Sudan	Quantitative; questionnaire.	Sample size: 33 participants; 10 surveillance officers, 10 community health workers, 5 health facility-based respondents, 4 senior-level county and central personnel, 2 laboratory-based respondents, and 2 data managers.
Ario et al. (76)	Uganda	Quantitative; semi-structured questionnaires.	Sample size: 293 participants; clinicians, nurses, records assistants, laboratory and environmental health personnel. Roles in IDSR include health facility leaders, surveillance focal persons, and village health team members.
Kambalame et al. (79)	Malawi	Qualitative; case study, focus group discussion.	Sample size: 43 participants; focal persons for various programs and surveillance systems, data clerks, facility in-charges, senior clinical officers, representatives of Ministry of Health, participants from animal health and international organizations.
Zalwango et al. (80)	Uganda	Mixed methods; semi-structured questionnaire, key informant interviews, focus group discussion.	46 private and 8 public health facilities from three sub-counties. Additionally, 12 surveillance officers, a regional epidemiologist, two health facility surveillance officers, five health workers. six villages (FGDs).
Ibrahim et al. (81)	Nigeria	Quantitative; checklist, questionnaire.	Sample size: 34, participants; state epidemiologists, State Disease Surveillance and Notification Officers (SDSNOs) and their assistants, LGA DSNOs, health facility surveillance focal persons, clinicians, WHO cluster consultants, and WHO LGA technical facilitators.
Nyenswah et al. (75)	Liberia	Qualitative; interviews.	Sample size: 29 participants; age: median age category 40–44 years, range 30–65 years, sex: 17 males (58.6%), 12 females (41.4%). Cadre/Profession; medical doctors, public health experts, field epidemiologists, community members, directors of programs.
Stolka et al. (82)	Democratic Republic of Congo	Quantitative; questionnaire.	Sample size: 79 participants; health information officers, chief medical officers, medical officers in charge of disease surveillance, head physicians, head nurses, and community health workers.
Adokiya et al. (83)	Ghana	Qualitative; key informant interviews.	Sample size: 18 participants; sex: 12 males, 6 females; Cadre/profession: 7 disease control officers, 4 physician/medical assistants, 3 general staff nurses, 1 community health nurse, 1 biomedical scientist, 1 health information officer, 1 nutrition officer.
Twene et al. (84)	Ghana	Quantitative; structured questionnaire, observation, review of secondary data.	Sample size: 347 health care workers. Participants; age 25–30 years: 42.7%, 31–35 years: 34.9%, 36–40 years: 15.3%, 41–45 years: 3.5%, 45 and above: 3.8%; Sex Male: 38.0%, Female: 62.0%
Nagbe et al. (85)	Liberia	Quantitative; checklist, secondary data, document review.	Sample size 384 participants; district surveillance officers (DSOs), zonal surveillance officers (ZSOs), and officers in charge (OICs) at health facilities.
Benson et al. (86)	South Africa	Quantitative; semi-structured questionnaire.	Sample size: 114 participants; median age: 49 years (range: 26–69 years). Roles: disease control and response (53%), disease detection, health management, others. Most participated in NORT (43%). Median NDSS experience: 11 years. Median training duration: 2 weeks.
Siya et al. (87)	Uganda	Mixed methods; semi structured questionnaire, focus group discussions.	Sample size: 48 Participants; age: all participants were adults (18 years and above); sex: not specified; village health team (VHT) professionals, part of community health care workers at Health Center I.
Mwatondo et al. (88)	Kenya	Quantitative; structured questionnaire, observation.	Sample size: 175 partcipants; healthcare worker responsible for surveillance or officer-in-charge of reporting activities at each facility.
Jinadu et al. (89)	Nigeria	Mixed methods; questionnaire, key informant interviews.	Sample size: 531 participants; mean age: 42 ± 8.1 years, gender: 86.1% female; predominantly community health extension workers (CHEWs) at 36.9%.
Beebeejaun et al. (90)	Nigeria	Mixed methods; document reviews, observations, and questionnaires.	Sample size: 19 participants; call handlers, surveillance officers, data management staff, department heads, NCDC senior leadership.
Chimsimbe et al. (91)	Zimbabwe	Mixed methods; questionnaires, checklists, key informant interviews.	Sample size: 46. Sex: female (83%), registered general nurses (65%), primary care nurses, and medical doctors, median years in service: 10 years (Q1 = 3, Q3 = 14).
Ssendagire et al. (92)	Somalia	Mixed methods; document review, online survey, and key informant interviews.	Sample size; 21 participants; key persons involved in the planning and implementation of IDSR.
Nakiire et al. (30)	Uganda	Qualitative; focus group discussion.	Sample size: 216 participants; mean age: 38.8 years, gender: 58% female, profession: 47% nurses (*n* = 101), other professions include clinicians, laboratory staff, medical records officers, and others.
Nakiire et al. (30)	Kenya	Mixed methods, secondary data, field visits, key informant interviews, focus group discussions.	Sample size 2,343 in Siaya, 3,100 in Nakuru, and 305 in Marsabit trained for CEBS, and 288 healthcare workers trained for HEBS across Nakuru, Mombasa, and Meru counties. community health volunteers (CHVs), community health assistants (CHAs), and healthcare workers (HCWs).
Mremi et al. (32)	Tanzania	Quantitative; mapping, in-depth interviews, and desk reviews.	Sample size: 21 CHMT members; Age: median 44.5 years (IQR 37-53); Sex: 10 females, 11 males; education: university education (Bachelor‘s: 7, Master's: 7), advanced diploma: 3, ordinary diploma: 3, secondary school: 1; role in IDSR: Members of CHMT responsible for overseeing health activities and data management in IDSR.
Yusuf et al. (93)	Ethiopia	Quantitative; questionnaire.	Sample size: 297, health professional, age: median age is 28 years; 30.64% are aged 25–28 years, sex: 61.95% male, majority are nurses (52.53%), position: 32% have administrative experience, nurses, medical doctors.
Lakew et at. (94)	Ethiopia	Quantitative; questionnaires, document reviews, observations, and discussions with key personnel.	82 reporting units including five zones, two town administrations, 21 woredas, and 54 health facilities. six WHO surveillance officers and six government surveillance counterparts.
Martin et al. (95)	Sierra Leone	Quantitative; questionnaire, observation.	Sample size: 47 users, their role in IDSR involves using the eIDSR system for data entry and reporting.
Kallay et al. (96)	Democratic Republic of Congo	Mixed methods, questionnaire, observations, focus group discussion, open ended questions.	Sample size: 72 participants; medical biologist, medical doctor, nurse A1, nurse A2, nurse A3, nurse L3.
Adokiya et al. (97)	Ghana	Mixed methods; secondary data, key informant interviews.	Sample size: 6 participants; sex: all male, health system workers with training in disease surveillance, involved in disease surveillance and health information units.
Saleh et al. (31)	Zanzibar	Mixed methods; document review, observations, structured interviews.	Sample size: 57 participants; 45 health facility in-charges or designated disease surveillance staff, 10 district surveillance officers, 2 staff from the epidemiology and HMIS units.
Mandyata et al. (33)	Zambia	Qualitative; key informant interviews.	Sample size 13 participants; IDSR specialist, monitoring and evaluation officer, disease surveillance unit officers, health information unit officers, laboratory Officers-in-charge, and medical/nursing officers-in-charge.
Wu et al. (35)	Malawi	Mixed methods; secondary data, interviews, focus group discussions, and observations.	Sample size: 36 participants, age: 28–52 years old, sex: majority male (22 males), health surveillance assistants (HSAs), health care workers (HCWs), district health management team (DHMT) officers, Ministry of Health.
Issah et al. (98)	Ghana	Mixed methods; in-depth interviews, document review, checklist, secondary data.	Sample size 18. Age range: 4–54 years, sex: 14 males, 7 medical directors, 2 district directors, over 20 years in the health system.
Lafond et al. (99)	Nigeria	Quantitative; questionnaire.	Sample size: 245 physicians. Participants, Median age: 37 years (range 28–62). Gender: 67% male, public sector physicians (pediatrics, internal medicine, general practice), frontline for reporting notifiable diseases.
Toda et al. (100)	Kenya	Quantitative; questionnaire.	Sample size: 142 participants (131 health facility in-charges and 11 sub-county disease surveillance coordinators), age: median age of disease surveillance coordinators is 44 years, sex: nearly all disease surveillance coordinators were male (90.9%).
Ng'etich et al. (16)	Kenya	Mixed methods; questionnaire.	Sample size: 192 health facility workers, age: 61% aged between 18 and 40 years, Sex: 51% female, Cadre: 65% nursing, 22% clinical, 10% public health staff, responsible for surveillance data collection, collation, and transmission.
Masiira et al. (101)	Uganda	Mixed methods; questionnaire, checklist, focus group discussions, key informant interviews.	Sample size: 606 health workers: Participants; 391 clinicians and nurses, 108 laboratory workers, 81 District Health Team members, and 26 district laboratory focal persons, 26 District Health Officers and six national-level program managers or department heads participated as key informants. The sample was selected from 26 districts across 13 health regions in Uganda, focusing on those who had undergone IDSR training.
Girdler-Brown et al. (102)	South Africa	Quantitative	Not mentioned; the paper focuses on data sets from surveillance systems rather than individual participants.
Njeru et al. (74)	Kenya	Quantitative; questionnaire.	Sample size: 78 surveillance officers. Participants; 60 sub county surveillance coordinators, 10 county surveillance coordinators, 8 county health records and information officers.
Ng'etich et al. (36)	Kenya	Mixed methods; questionnaire, checklist, interview.	Sample size: 283 participants; age: majority over 30 years, sex: 58% male, community health workers, health facility workers, sub-county and county level personnel, education; Health personnel with at least a diploma or higher education.
Onwe et al. (103)	Nigeria	Mixed methods; questionnaire.	Sample size: 14 participants; 50% male, 50% female, 50% State epidemiologists, 50% Disease Surveillance and Notification Officers (DSNOs).

### 2.5 Risk of bias (quality) assessment

We used Dearholt and Dang's Johns Hopkins Nursing Evidence Appraisal Tool (17) to assess the quality of the included studies as exemplified by Ng'etich et al. (16). Quality was assessed based on the strength of evidence (Levels I-V) and the quality of evidence (Grades A, B, and C).

The strength of evidence was assigned to Levels I, II, III, IV, or V, depending on whether the report was based on an experimental design, quasi-experimental design, non-experimental design, expert opinion based on research evidence, or expert opinion based on non-research evidence. Each eligible study was assigned a grade of A, B, or C depending on whether the quality of the research evidence was high, good, or low. Findings from studies with lower levels of evidence or quality were assessed more critically. Please refer to Supplementary File 3 as a detailed guide. Two reviewers independently completed this task, and any disagreements were resolved by consensus. If a consensus was not reached, disputes were resolved by the senior author ASM.

### 2.6 Data and sensitivity analysis

Our decision to synthesize the extracted data was informed by the relevance of the included studies, rather than the sheer number of available studies. For quantitative studies, we did not conduct a meta-analysis due to heterogeneity of data, and as such, the data were analyzed together with qualitative data using the directed content analysis (18). First, we created a coding scheme from the predetermined codes. We used CFIR where its constructs/subconstructs were utilized as predetermined codes. We selected the CFIR as a determinant framework because it provides a taxonomy, codebook, and clear construct definitions that fit well with different health contexts. The updated CFIR comprises 67 constructs organized into five domains: Innovation domain, Outer setting, Inner Setting, Individuals domain, and Implementation process domain (19). We operationalized the CFIR constructs using the developers' guide to inform the development of a structured coding framework where we contextualized the coding framework by defining the subject of each domain for the intervention: We defined Innovation as the IDSR system and its associated tools, the Outer Setting was defined by national guidelines including reporting mandates and accountability to the next higher levels, Inner Setting was operationalized at both facility and district levels. The Individuals domain captured the perspectives of the primary implementers and their supervisors. The process domain encompassed activities carried out to implement the IDSR. The code book developed by the developers of the CFIR was used as a guide for defining constructs and coding definitions.

Second, determinants in the included texts were coded either as a barrier or a facilitator. The determinants were coded as facilitators if their presence promoted implementation else, the determinants were coded as barriers if they hindered the implementation, as a result, the excerpts from the included texts were categorized into barriers and facilitators.

Third, the barriers and facilitators were mapped to CFIR constructs/subconstructs according to the coding scheme and then categorized into the five CFIR domains (18, 19). The frequency of statements (coded excerpts) on the constructions was counted. The frequency of the statements on the construction does not necessarily reflect the relative importance or causal weight of a barrier or facilitator.

Fourth, Inductive thematic analysis (20) was conducted separately within each CFIR domain. CFIR-coded barrier and facilitator excerpts were first synthesized into concise summary statements, which were then iteratively merged into higher-order themes within their respective domains based on conceptual similarity and recurrence across studies. The initial thematic analysis was undertaken by one reviewer and subsequently reviewed by other members of the review team, with refinements made through discussion to ensure coherence and analytic consistency. CFIR offers a systematic theoretical approach for identifying barriers and facilitators of intervention implementation (19). We did not perform a subgroup analysis. The findings of this review were reported in accordance with the PRISMA guidelines (15).

The reviewers had comprehensive training in the updated CFIR. TMA organized training for the reviewers which was facilitated by implementation research experts from Norway. In addition, the first and the senior authors have experience in facilitating implementation research short courses for healthcare workers in Malawi, making them have a good understanding of the framework.

Two reviewers independently completed the CFIR coding process, and any disagreements were resolved by consensus. If consensus was not reached, the senior author ASM, was engaged to resolve the disputes.

## 3 Results

### 3.1 Description of the study selection process

**Figure 1:**
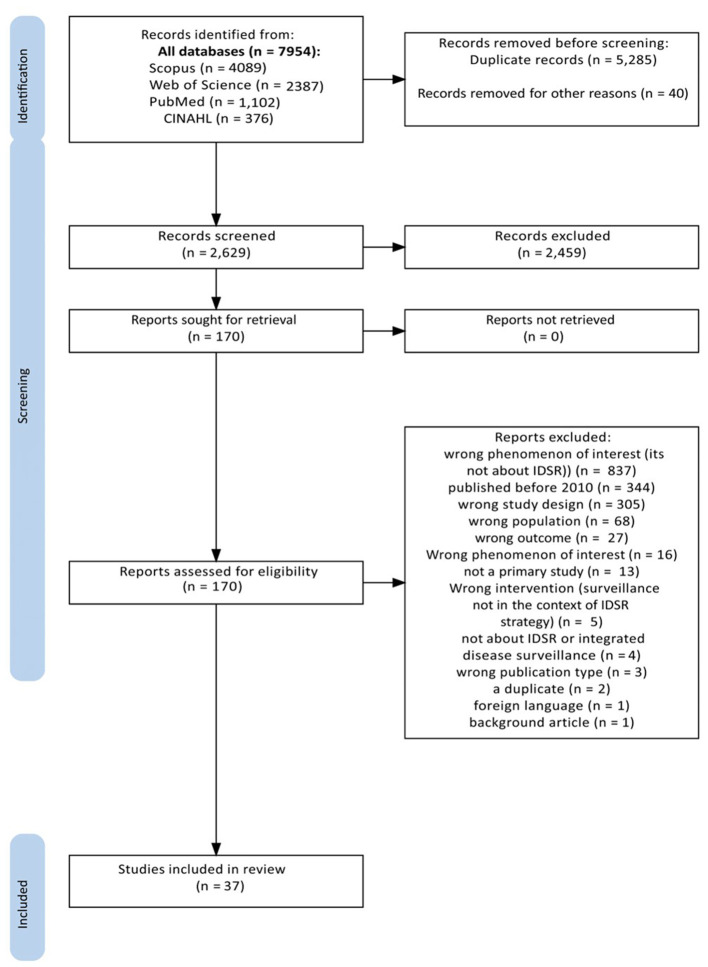
Prisma flow diagram showing the process of identifying studies from electronic databases. A total of 37 studies were included in the review.

Figure 1 shows a PRISMA flow diagram illustrating the study selection process. A comprehensive search across four databases, Scopus, Web of Science, MEDLINE via PubMed, and CINAHL, resulted in 7,954 articles. Of these, 5,284 duplicates were eliminated. Prior to title and abstract screening, the first author excluded 40 studies, as they were conference proceedings. Subsequently, 2,459 articles were removed following title and abstract screening conducted by two research team members, DMK and JM. Of the 170 studies for which the full texts were reviewed, 37 met the eligibility criteria and were included in the final review.

### 3.2 Quality (risk of bias) assessment of included studies

Table 1 summarizes the quality assessment of the included studies. A detailed assessment of the quality of each study is provided in Supplementary File 2. Most studies were assigned to evidence level III according to Dearholt and Dang's Johns Hopkins Nursing Evidence Appraisal Tool (18) because of their non-experimental study designs (*n* = 36). One study received level II evidence to employ a quasi-experimental design (19). Most studies (59.5%) were classified as having low quality evidence (grade C) based on their methodological approach (Table 1). Only two studies (20, 21), representing 5.4% of the included studies, were deemed to have high-quality evidence (grade A). No studies were excluded from the analysis based on evidence strength and quality, and all studies meeting the inclusion criteria were included in the analysis.

#### 3.2.1 Overview of the characteristics of included studies

Tables 1A–C present a summary of the 37 studies reviewed, categorized by study type, country of implementation, participant groups, research aims, and the frameworks employed. Most of the studies (*n* = 27) utilized mixed methods approaches, while six employed quantitative methods and five used qualitative methodologies (Table 1A). Geographically, the studies were predominantly conducted in Kenya (*n* = 6), followed by Uganda, Nigeria (*n* = 5 each), and Ghana (*n* = 4). Other countries included the Democratic Republic of Congo (DRC), Ethiopia, Malawi, Liberia, and South Africa, with two studies each, and Sierra Leone, Somalia, South Sudan, Tanzania, Zambia, Zanzibar, and Zimbabwe, with one study each. One study encompassed multiple countries, including the Ivory Coast, Ecuador, Madagascar, Namibia, and the Kingdom of South Arabia, however, our analysis focused on the results reported from African countries, as we focused on the results from Ivory Coast, Madagascar and Namibia (Table 1A).

**Table 1B:** Study objectives and frameworks.

**Author (Year)**	**Primary study objective**	**Framework used**
Meierkord et al. (77)	To investigate approaches to capacity building and training for disease surveillance at the national level, understand the potential role of National Public Health Institutes (NPHIs), identify challenges and opportunities to improve disease surveillance, identify core areas where disease surveillance	Not mentioned
Rumunu et al. (78)	To evaluate the performance of the Integrated Disease Surveillance and Response (IDSR) and Early Warning and Response Network (EWARN) systems at national, state, county, health facility, and community levels in South Sudan during the first half of 2021. The study aimed to describe these systems, assess their performance	WHO protocol and the guidelines for evaluating early warning alert and response networks
Ario et al. (76)	To evaluate the public health surveillance system in refugee settlements in Uganda and to improve public health practice and disease control activities, specifically for epidemic or endemic diseases.	US CDC Updated Guidelines and the Technical Guidelines for IDSR
Kambalame et al. (79)	To explore the perspectives of key surveillance players across healthcare levels regarding factors influencing the operationalization of Integrated Disease Surveillance (IDS) in Malawi, as part of a larger multi-country study to identify context-specific factors affecting IDS operationalization.	International Association of Public Health conceptual framework
Zalwango et al. (80)	To explore and investigate gaps in surveillance that may have resulted in the late detection of the Sudan virus disease (SVD) outbreak in Uganda, focusing on both systemic and knowledge-related gaps in the Integrated Disease Surveillance and Response (IDSR) system.	Not mentioned
Ibrahim et al. (81)	To conduct a rapid assessment of the Integrated Disease Surveillance and Response (IDSR) system in three northeastern states of Nigeria (Adamawa, Borno, and Yobe) to identify and address gaps, collect best practices, and identify areas for improvement to strengthen the system.	WHO-AFRO assessment protocol for national disease surveillance systems and epidemic preparedness and response
Nyenswah et al. (75)	To describe the successes, failures, strengths, and weaknesses in the development, adoption, and implementation of Liberia's Integrated Disease Surveillance and Response (IDSR) strategy from 2004 to early 2014, and to explore factors that accelerated or hindered its development, as well as why the health	Not mentioned
Stolka et al. (82)	To assess the surveillance system for priority zoonotic diseases in Kinshasa and Haut Katanga provinces of the DRC, identify priority activities for strengthening the surveillance system, and develop an improvement plan to address weaknesses in the system.	Not mentioned
Adokiya et al. (83)	To assess the core and support functions of the Integrated Disease Surveillance and Response (IDSR) system at the periphery level of the health system in northern Ghana.	Core and Support functions of IDSR
Twene et al. (84)	To to assess the factors influencing the Integrated Disease Surveillance and Response (IDSR) system in selected districts in the Eastern Region of Ghana, with the goal of strengthening its implementation and enhancing the region's ability to detect and prevent disease outbreaks.	WHO protocol for the assessments of national communicable disease surveillance
Nagbe et al. (85)	To describe the different approaches used for the implementation of Integrated Disease Surveillance and Response (IDSR) in Liberia from 2015 to 2017, including innovations, best practices, and lessons learned after the Ebola Virus Disease outbreak.	Not mentioned
Benson et al. (86)	To determine the perceptions of key stakeholders on the national NDSS attributes of acceptability, flexibility, simplicity, timeliness, and usefulness.	US Centers for Disease Control and Prevention (CDC) Updated Guidelines for Evaluating Public Health Surveillance Systems
Siya et al. (87)	To explore the capacities of village health teams (VHTs) in Sebei communities of Mount Elgon in undertaking surveillance tasks for emerging and re-emerging infectious diseases in the context of a changing climate, and to identify shortcomings in disease-reporting infrastructure to improve public health	Not mentioned
Mwatondo et al. (88)	To determine the prevalence of adequate reporting and factors associated with IDSR reporting among health facilities in Nairobi County, Kenya. Specifically, the study aimed to review the extent of weekly reporting for IDSR priority diseases, determine the timeliness and completeness of these reports.	WHO protocol and the IDSR Technical guidelines
Jinadu et al. (89)	To assess the knowledge and attitude of healthcare workers toward the Integrated Disease Surveillance and Response (IDSR) strategy for epidemic-prone diseases at the primary health care level in Oyo State, Nigeria, and to determine the associated factors influencing this knowledge and attitude.	WHO/Centre for Disease Control (CDC) protocol for communicable disease surveillance system monitoring
Beebeejaun et al. (90)	To describe the NCDC EBS system and the nature of signals and events detected; evaluate the system against its objectives; and provide recommendations to improve effectiveness, efficiency, and maximize utility of the system.	CDC guidelines for the evaluation of public health surveillance systems
Chimsimbe et al. (91)	To evaluate the Notifiable Disease Surveillance System (NDSS) in Chegutu District to determine reasons for its underperformance and data discrepancies, and to provide evidence-based recommendations for improving the system.	Centres for Disease Control and Prevention (CDC) Update Guidelines for Evaluating Public Health Surveillance Systems.
Ssendagire et al. (92)	To report on the progress and experiences of implementing an integrated disease surveillance and response system (IDSRS) in Somalia between 2016 and 2023, and to explore factors contributing to this progress. The findings are intended to be useful for other member states in the region aiming to accelerate	Not mentioned.
Nakiire et al. (30)	To explore health workers' perceptions regarding the revitalized Integrated Disease Surveillance and Response (IDSR) training in Uganda, specifically assessing the benefits, constraints, and suggestions for improvement of the training.	Not mentioned
Nakiire et al. (30)	To describe the modifications and expansion of Kenya's event-based surveillance (EBS) system to include COVID-19-related signals, demonstrate how the existing EBS structure was adapted to detect COVID-19 cases, describe the infrastructure of community event-based surveillance (CEBS) before the pandemic	Not mentioned
Mremi et al. (32)	To assess the capacity to manage and utilize disease surveillance data and implement an intervention to improve data analysis and use at the district level in Tanzania.	Not mentioned
Yusuf et al. (93)	To determine the level of integrated disease surveillance response (IDSR) practice and associated factors among health professionals in the West Hararghe zone, eastern Oromia, Ethiopia.	Not mentioned
Lakew et at. (94)	To assess the performance of the disease surveillance and immunization system in Amhara Region, Ethiopia, with emphasis on low-performing woredas and zones, focusing on VPD surveillance and its integration into PHEM, and to share experiences among WHO officers and their government counterparts.	WHO - AFRO tool for external surveillance review
Martin et al. (95)	To develop an Integrated Disease Surveillance and Response (IDSR) system, transitioning it from a paper-based to an electronic system (eIDSR), and demonstrating the feasibility of a national-scale mHealth-based surveillance system.	Not mentioned
Kallay et al. (96)	To evaluate the performance in core and supporting functions of the IDSR system in North Kivu Province Health Zones at risk of an EVD epidemic, to determine the system's capacity to identify, report, investigate and respond to epidemic prone diseases.	IDSR guidelines and the US CDC's Guidelines for Evaluating Public Health Surveillance Systems
Adokiya et al. (97)	To evaluate the Integrated Disease Surveillance and Response (IDSR) system in northern Ghana, focusing on its functioning and data quality as reported through the DHIMS2 network.	IDSR technical guidelines
Saleh et al. (31)	To assess the performance of the core and support functions of the Zanzibar integrated disease surveillance and response (IDSR) system to determine its capacity for early detection of and response to infectious disease outbreaks.	WHO guide and protocol for assessment of national communicable disease surveillance and response systems
Mandyata et al. (33)	To investigate and report on the existing challenges in the implementation of the Integrated Disease Surveillance and Response (IDSR) strategy in Zambia from a health worker perspective.	WHO Protocol
Wu et al. (35)	To understand the state of IDSR implementation and differences between guideline and practice for future disease surveillance system strengthening. It also aimed to describe the process of case identification and reporting in practice and explore the differences between the IDSR guideline and actual	IDSR technical guidelines
Issah et al. (98)	To assess the quality, core, and support functions of the integrated disease surveillance and response (IDSR) system and to identify gaps in its implementation relating to 18 suspected cases of Ebola virus disease (EVD) in the Brong Ahafo Region, Ghana.	Core and Support functions of IDSR
Lafond et al. (99)	To describe the knowledge, perceptions, and practices related to infectious disease reporting through the IDSR system, identify physicians' preferred sources of health information, and assess their knowledge of avian influenza infection in humans among public sector physicians in Nigeria.	Not mentioned
Toda et al. (100)	To assess the knowledge of Integrated Disease Surveillance and Response (IDSR) standard case definitions among health workers and their supervisors in rural Kenya.	Not mentioned
Ng'etich et al. (16)	To evaluate surveillance system attributes based on healthcare workers' perceptions in relation to preventive chemotherapy-targeted neglected tropical diseases (PC-NTDs) endemic in Kenya.	CDC guidelines for the evaluation of public health surveillance systems
Masiira et al. (101)	To assess the core and support functions of the Integrated Disease Surveillance and Response (IDSR) after the implementation of the revitalized IDSR programme in Uganda, and to document whether the programme was implemented adequately.	Not mentioned
Girdler-Brown et al. (102)	To compare the performance of the South African National Disease Surveillance System (NDSS) with the infectious disease laboratory data from the National Health Laboratory Services (NHLS) using five parameters: Completeness, Stability, Representativeness, Sensitivity, and Positive Predictive Value (PPV)	Not mentioned
Njeru et al. (74)	To evaluate the impact of training on the timeliness and completeness of IDSR reporting rates in Kenya's new DHIS2 platform and to identify challenges affecting surveillance reporting rates.	Not mentioned
Ng'etich et al. (36)	To assess the surveillance system core and support functions relating to preventive chemotherapy-targeted neglected tropical diseases (PC-NTDs) in Kenya, specifically focusing on Soil Transmitted Helminths, Schistosomiasis, Trachoma, and Lymphatic Filariasis.	WHO protocol for assessing national surveillance system and the CDC updated guidelines for evaluating public health surveillance systems
Onwe et al. (103)	To determine the existence and effect of vertical programs on disease surveillance and response in Nigeria.	Health system building blocks

**Table 1C:** Evidence level and quality ratings.

**Author (Year)**	Evidence level^*^	Quality grade^**^
Meierkord et al. (77)	II	B
Rumunu et al. (78)	II	C
Ario et al. (76)	II	A
Kambalame et al. (79)	II	B
Zalwango et al. (80)	II	C
Ibrahim et al. (81)	II	C
Nyenswah et al. (75)	II	A
Stolka et al. (82)	II	C
Adokiya et al. (83)	II	B
Twene et al. (84)	II	B
Nagbe et al. (85)	II	B
Benson et al. (86)	II	C
Siya et al. (87)	II	C
Mwatondo et al. (88)	II	B
Jinadu et al. (89)	II	B
Beebeejaun et al. (90)	II	C
Chimsimbe et al. (91)	II	C
Ssendagire et al. (92)	II	C
Nakiire et al. (30)	II	B
Nakiire et al. (30)	II	C
Mremi et al. (32)	II	C
Yusuf et al. (93)	II	B
Lakew et at. (94)		
Martin et al. (95)	II	C
Kallay et al. (96)	II	C
Adokiya et al. (97)	II	C
Saleh et al. (31)	II	C
Mandyata et al. (33)	II	B
Wu et al. (35)	II	B
Issah et al. (98)	II	B
Lafond et al. (99)	II	C
Toda et al. (100)	II	C
Ng'etich et al. (16)	II	C
Masiira et al. (101)	II	B
Girdler-Brown et al. (102)	II	C
Njeru et al. (74)	III	C
Ng'etich et al. (36)	II	C
Onwe et al. (103)	II	C

^*^Evidence levels reflect the hierarchy of study design (e.g., Level II = analytical observational or qualitative studies; Level III = descriptive or exploratory studies).

^**^Quality grades indicate methodological rigor (Grade A = high, Grade B = moderate, Grade C = low). These classifications describe the strength and quality of the evidence base and do not imply the magnitude, direction, or causal impact of reported implementation barriers or facilitators.

The participant profiles varied across studies, with surveillance officers being the most frequently involved (*n* = 24), followed by data clerks and clinicians (*n* = 15 each), nurses (*n* = 9), laboratory personnel and community health workers (*n* = 6 each), in-charge health facilities, and general healthcare workers (*n* = 5 each). Additional participants included epidemiologists, regional respondents, district management team members, and program focal persons (Table 1A).

In terms of research aims, 18 studies focused on evaluating surveillance systems, whereas 12 aimed to identify barriers and facilitators for the implementation of integrated disease surveillance and response (IDSR) systems. Four studies investigated the existing gaps and challenges, and two assessed the knowledge, perceptions, and practices of surveillance practitioners. Other objectives included evaluating the feasibility of scaling national surveillance systems and comparing the surveillance system performance with laboratory data (Table 1B).

### 3.3 CFIR domains and barrier constructs

**Figure 2:**
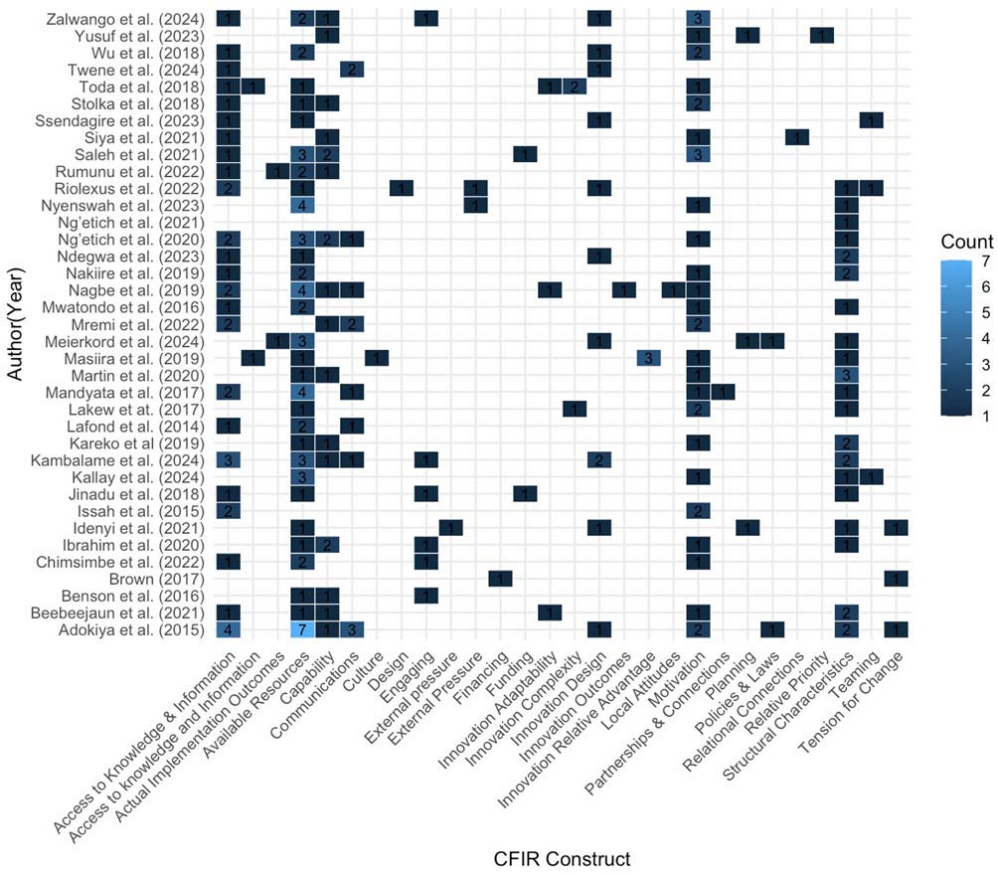
Heat maps showing the distribution of coded CFIR constructs across included studies. Cell values represent counts of coded excerpts (*n* = coded excerpts), illustrating the relative prominence of CFIR barriers reported in the literature. Darker shading indicates higher coding density.

Thirty-four constructs (*n* = 34) from six domains were identified as barrier determinants. Table 2 summarizes the barrier results for the CFR domain and associated constructs, and Figure 2 shows the distribution of coded CFIR constructs across included studies. Reported frequencies represent the number of extracted and coded excerpts mapped to each CFIR construct across all included studies. The inner setting of the health facility itself emerged as a predominant barrier to the implementation of the IDSR. Studies have reported several key elements within the internal setting, including available resources (*n* = 64), access to knowledge and information (*n* = 33), and structural characteristics (*n* = 30). The individual domain was also a crucial barrier (*n* = 67), with motivation (*n* = 45), and capability (*n* = 22) being the only constructs identified.

**Table 2:** Barrier results by CFIR domains and constructs.

**CFIR domain**	**Constructs**	**Barrier statement**	**Exemplar quotes (Author, Year of Publication)**
Innovation domain	Innovation adaptability *n* = 4 Innovation complexity *n* = 4 Innovation design *n* = 12 Innovation relative advantage *n* = 2	Low level of surveillance system flexibility Difficulty in understanding IDSR related materials and completing basic tasks Lack of integration in reporting surveillance data to the next level, Unclear data sources, Limited scope i.e. surveillance system, case definitions IDSR is inferior to laboratory surveillance system.	Lack of flexibility and stability in the surveillance system (28) Challenges with completing IDSR forms and understanding guidelines (28) Verticalization leading to duplication and coordination challenges (16) The NDSS underperforms compared to the laboratory system in terms of completeness, stability, and representativeness (30).
Outer setting domain	External pressure *n* = 3 Financing *n* = 1 Local attitudes *n* = 2 Policies and laws *n* = 2	undue donor influence in surveillance activities Lack of external budget support Stakeholder negative perception about IDSR Lack of data sharing facilitating frameworks, Failure to comply with international standards for case confirmation.	Parallel reporting systems driven by Implementing Partners (3) Lack of budget support from government (3) Negative perceptions from experienced stakeholders and those in disease detection (12) Challenges in international data sharing due to dependency on personal initiatives and negative consequences (1).
Inner setting domain	Access to knowledge and information *n* = 33 Available resources *n* = 64 Funding *n* = 15 Materials and equipment *n* = 16 Space *n* = 1 Communications *n* = 11 Culture *n* = 1 Mission alignment *n* = 3 Partnerships and connections *n* = 1 Relational connection *n* = 1 Relative priority *n* = 1 Structural characteristics *n* = 30 Information technology infrastructure *n* = 16 Work infrastructure *n* = 14 Tension for change *n* = 3.	Lack of or inadequate training, supervision, feedback and information materials Shortage of staff, high staff turnover, industrial action, lack of training, lack of designated IDSR focal person Lack of resources including funding, materials and equipment, transport, and physical space Lack of reporting system, inadequate feedback mechanisms, lack of communication between surveillance and HMIS officers perception that IDSR is a vertical program high reporting burden, high workload, competing tasks Difficulty in obtaining data from private health facilities. Lack of relational connection with other One-Health relevant Sectors high reporting burden, high workload, competing tasks Absence of interoperability of data systems, use of old data systems/software, unstable electronic data systems, poor network signal Absence of interoperability of data systems, use of old data systems/software, manual and paper based data systems, unstable electronic data systems, poor network signal Abuse of data capturing systems especially mobile devises Shortage of staff, high staff turnover, industrial action, lack of training, lack of designated IDSR focal person Low priority for disease surveillance activities, duplication of efforts.	Insufficient training of health workers on Integrated Disease Surveillance and Response (3) Lack of standardized laboratory reporting forms and equipment (2) Lack of case definitions and diagnostic codes (4) There is inadequate funding to support IDSR activities (11) Lack of feedback to those reporting (4) Perception of IDSR as a vertical program (29) Surveillance officers having other competing tasks (3) Difficulty in obtaining data from private health facilities (16) Lack of engagement in reporting environmental and wildlife-related hazards (13) Lack of data system interoperability (19).
Individuals domain	Capability *n* = 22 Implementation facilitator *n* = 2 Innovation deliverers *n* = 15 Motivation *n* = 45 Innovation deliverers *n* = 2 Innovation facilitators *n* = 6	Lack of knowledge and skills to perform surveillance functions i.e. data management and analysis, supervision, feedback Lack of knowledge about case definitions and guidelines Inconsistent supervision from implementation facilitators, and inadequate training for surveillance deliverers Delays in sending data to the next higher level in time Lack of commitment to conduct community based and active surveillance, carryout response activities, and properly manage data Low acceptability of IDSR, Poor adherence to IDSR Materials especially Guidelines and case definitions, under-recognition of priority diseases, underutilization of laboratory for confirmation of cases Poor data and reporting completeness In-consistent supervision	Limited capacities of personnel to identify, report, analyse, and interpret IDSR data (6) Low knowledge of case definitions among health workers (8) Infrequent supervision from higher-level health authorities (8) Poor timeliness of reports (24) Poor timeliness of reports (74) Low scores for the NDSS in acceptability (30) Health facilities not sending surveillance data (3) Weak community engagement and staff supervision (7)
implementation process	Engaging *n* = 7 Innovation deliverers *n* = 1 Planning = 3 Teaming =3	Lack of involvement of key stakeholders in reporting surveillance data especially private health facilities and other One-Health sectors Lack of adequate planning i.e. training, surveillance focal points terms of reference Poor coordination of surveillance activities leading to verticalization limited functionality of RRTs	Poor private sector involvement in surveillance (5) Lack of a national training agenda (1) Limited functionality of RRTs due to high turnover and lack of standardization (75)
Outcomes	Innovation outcomes *n* = 2 Deliverer Impacts *n* = 2	Perceived low usefulness	Low scores for the NDSS in usefulness (30)

### 3.4 CFIR domains and facilitator constructs

**Figure 3:**
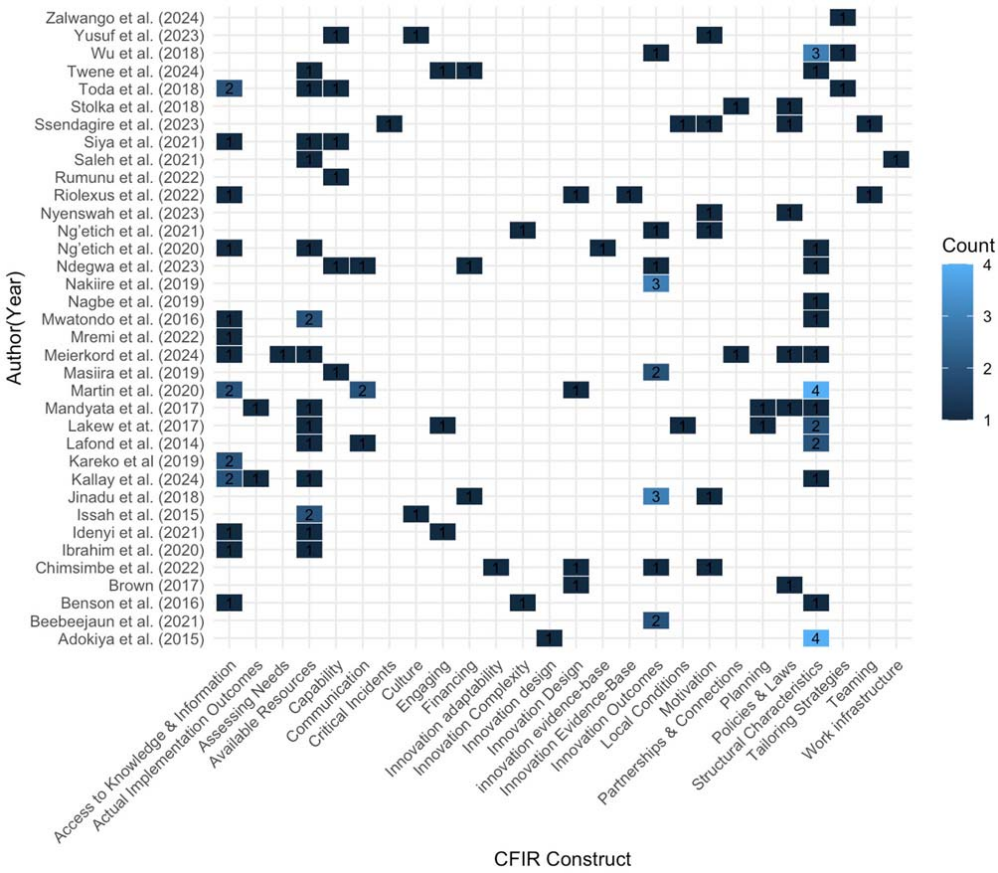
Heat maps showing the distribution of coded CFIR constructs across included studies. Cell values represent coded excerpts (*n* = coded excerpts), illustrating the relative prominence of CFIR facilitators reported in the literature. Darker shading indicates higher coding density.

Twenty-five constructs (*n* = 25) from six domains were identified as enablers. Table 3 summarizes the enabler results organized by the CFR domains and constructs and Figure 3 shows the distribution of coded CFIR constructs across included studies. The inner setting of the health facility also emerged as a predominant enabler affecting the implementation of the IDSR. The most frequently identified structural characteristics (*n* = 24) were access to knowledge and information (*n* = 18) and available resources (*n* = 17). The outcome domain was also a crucial enabler, with innovation outcomes (*n* = 15) as the most identified construct.

**Table 3:** Facilitator results by CFIR domains and constructs.

**CFIR domain**	**Constructs**	**Facilitator statement**	**Exemplar quotes (Author, Year of Publication)**
Innovation domain	Innovation adaptability *n* = 1 Innovation complexity *n* = 4 Innovation design *n* = 5 Innovation evidence base *n* = 2	Flexibility to adapt to new information needs Simplicity of the IDSR system Integrated reporting systems including laboratory results, user friendly Resilience of the IDSR system Evidence of good performance in detecting, reporting, analysis	Flexibility to adapt to new information needs (17) Perception of simplicity, acceptability, and usefulness by some respondents (28) Including laboratory results in the notification system could improve data completeness and representativeness (30) High performance in specimen handling, report submission, and data analysis at sub-county and county levels (31)
Outer setting domain	Critical incidents *n* = 1 Financing *n* = 3 Local conditions *n* = 2 Partnerships and connections *n* = 2 Policies and laws *n* = 6	COVID-19 provided an opportunity for strengthening the health system Availability of funding from partners such WHO Leveraging foundations from other initiatives Presence of partnerships and connections with government and international partners for improved surveillance system and data sharing Adoption of IDSR guidelines, having policies for data sharing, and inclusion of laboratory data into IDSR system	Opportunity provided by the COVID-19 pandemic (16) Provision of resources by WHO at district and county levels (10) Existing foundation from EWARN system (16) Commitment from government and international partners to improve the surveillance system (8) Adoption of WHO guidelines to the Zambian context (16)
Inner setting domain	Access to knowledge and Information *n* = 18 Available resources *n* = 17 Funding *n* = 5 Materials and equipment *n* = 9 Communications *n* = 4 Culture *n* = 2 Deliverer-centeredness *n* = 2 Structural characteristics *n* = 24 Information technology infrastructure *n* = 18 Work infrastructure *n* = 6	Availability of information materials i.e. guidelines, case definitions, information dissemination platforms Improving knowledge and skills of staff through training, supervision and feedback Having adequate dedicated funding for surveillance activities having IDSR information materials (IDSR guidelines, case definition, reporting forms, posters) available at the facilities available diagnosis and response materials and equipment Availability of other materials such as cell phones and stationery improving interoperability of systems having continuous feedback mechanisms and electronic information dissemination solutions Having a designated surveillance focal person	Availability of IDSR technical guidelines and standard case definitions in all offices (though not effectively used) (18) Presence of trained surveillance focal points in many health facilities and health zone offices (75) Increasing budget allocations for workforce expansion (1) Availability of integrated case definitions and national PHEM guidelines (74) Presence of action thresholds for priority diseases (23) Availability of stationery for training and dissemination purposes (13) Development of systems to link the EBS dashboard to emergency operations canters (19) Continuous-feedback design addressing usability and functionality issues (76) Presence of trained surveillance focal points in many health facilities and health zone offices (75)
Individuals domain	Capability *n* = 6 Innovation deliverers *n* = 2 Motivation *n* = 9 High-level leaders *n* = 1	Having good knowledge of IDSR such as case definitions, thresholds, and how to use electronic gadgets Dedicated, committed health workers and stakeholders with positive attitude toward IDSR Improved completeness and timeliness of reporting Commitment of higher-level facilities to meeting reporting thresholds	Adequate knowledge of case definitions and alert thresholds among respondents (2) High-level commitment and stakeholder consensus (16) Improved completeness and timeliness of reporting (18) Higher-level facilities meeting reporting thresholds (28)
Implementation process	Engaging *n* = 2 Innovation deliverers *n* = 1 Planning = 4 Teaming = 4 Tailoring strategies *n* = 5	Integration of private health facilities and vertical programs into IDSR system Presence of plans especially training agenda, work plans, epidemic preparedness and well defined roles for staff Identifying strategies to overcome implementation barriers Presence of functional rapid response teams and technical working group for surveillance	Integration of private health care facilities into the IDSR system (10) Established IDSR implementation structure with defined roles (16) Use of community volunteers for case identification (24) Presence of rapid response teams and budget lines for epidemic response (23)
Outcomes	Actual implementation outcome *n* = 2 Adoption *n* = 2 Innovation outcomes *n* = 15 Deliverers impacts *n* = 6 Key decision maker impacts *n* = 1 Recipient impacts *n* = 6	Adoption of IDSR system to all health facilities perceived improvement in index of suspicion, reduction in morbidity and mortality, usefulness, data analysis, simplicity and acceptability Improved notification of priority diseases Innovation outcomes leading to improvement of population health: prevention of outbreaks, better detection of public heath events, improved response, and decrease in case fatality rate Innovation outcomes useful for strengthening the Community level Surveillance.	Integration of more health facilities into the IDSR system (75) Improved index of suspicion among HCWs (15) Consistent notification of COVID-19-related signals even during healthcare worker strikes (19) Enhanced response to disease outbreaks (18) Potential for strengthened community-level surveillance (24)

### 3.5 Themes of barriers and facilitators

**Figure 4:**
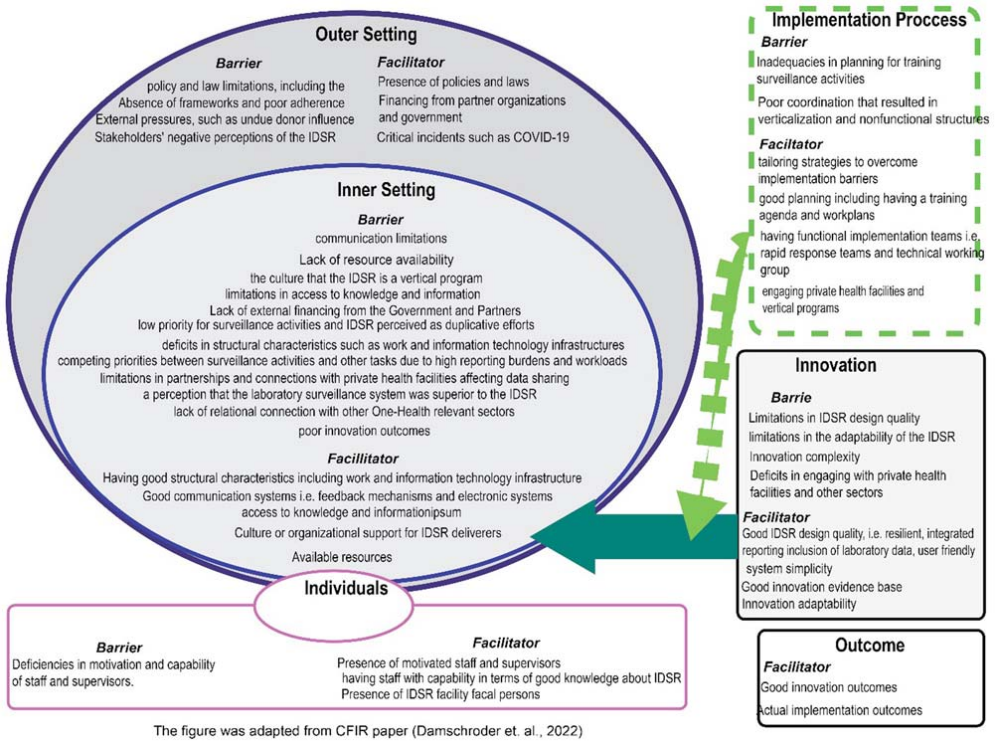
An overview of barrier and facilitator themes organized by the updated consolidated framework for implementation Research (CFIR) domains.

Barrier and enabler statements were generated based on CFIR barriers and enablers. Twenty-four themes (*n* = 24) related to barriers and facilitators were merged from the statements, as shown in Figure 4.

### 3.6 Interactions among CFIR constructs

Available resources interacted closely with access to knowledge and information, as limited funding, logistics and staffing constrained opportunities for training, supervision, and dissemination of surveillance guidance. In turn, Access to Knowledge and Information shaped Capability, with inadequate training and unclear guidance reducing health workers' ability to apply case definitions, conduct data analysis, and report priority conditions accurately. Weak Communication, particularly limited feedback from higher levels, interacted with Motivation, reducing willingness among health workers to prioritize surveillance and activities and sustain timely reporting. In addition, Innovation Design interacted with Work Infrastructure, whereby parallel reporting systems, complex forms, paper-based tools increased reporting burden and reduced system usability. Policies and Laws shaped Planning Processes by providing formal mandates and guidance for surveillance activities, although gaps in enforcement limited their effectiveness. Collectively, these findings demonstrate that implementation challenges arise from interconnected determinants operating across CFIR domains.

## 4 Discussion

This study aimed to investigate the barriers and facilitators of IDSR implementation in Africa. Previous reviews have largely focused on descriptive assessments of surveillance system attributes or on reporting implementation gaps without a unifying theoretical framework. While these studies have been valuable in characterizing system performance, they have provided limited insight into why implementation challenges persist across settings and how contextual and organizational factors interact to shape implementation outcomes. The current study used CFIR to systematically analyze the barriers and facilitators of IDSR implementation in Africa. CFIR enables systematic categorization of implementing determinants across multiple levels of the health system and supports analytic interpretation of how contextual, organizational, and individual factors jointly influence implementation experiences. To the best of our knowledge, this is the first study to synthesize evidence regarding IDSR implementation using implementation research theories, models, and frameworks. Our study found that six CFIR domains and 24 constructs explained the determinants of IDSR implementation in Africa, suggesting that the determinants of IDSR implementation may be explained by the CFIR framework. These domains include the innovation domain, outer setting domain, inner setting domain, individuals domain, implementation process, and outcomes.

### 4.1 Barriers to the implementation of IDSR

This review identified several barriers to IDSR implementation. Lack of available resources was identified as the primary barrier. Additional significant barriers included limitations in access to knowledge and information, deficits in structural characteristics such as limitations in work and information technology infrastructure, and deficiencies in the motivation and capability of staff and supervisors.

Other barriers included communication limitations, suboptimal innovation design quality, deficits in engaging with private health facilities and other sectors, limitations in the adaptability of the IDSR system, innovation complexity, inadequacies in planning for training surveillance activities, poor coordination that resulted in verticalization and nonfunctional structures, low priority for surveillance activities and IDSR perceived as duplicative efforts (tension for change); external pressures, such as undue donor influence on surveillance activities, a perception that the laboratory surveillance system was superior to the IDSR, poor innovation outcomes, stakeholders' negative perceptions of the IDSR (local attitudes), policy and law limitations, including the absence of frameworks and poor adherence, the culture in which the IDSR is a vertical program, limitations in partnerships and connections with private health facilities affecting data sharing, and a lack of relational connection with other One-Health relevant sectors, competing priorities between surveillance activities and other tasks due to high reporting burdens and workloads, and a lack of external financing from government.

#### 4.1.1 Innovation domain

The IDSR system faces several implementation challenges related to innovation characteristics, including low adaptability to new information needs, complexity that hinders understanding and task completion, limited scope in disease coverage and case definitions, lack of integration across disease programs, and relative (dis)advantage that IDSR is inferior to systems such as laboratory surveillance systems. These findings are in line with the previous literature that innovation adaptability, complexity, design, and relative advantage influence innovation implementation in the health sector (22, 23). Unlike similar systematic reviews of IDSR that largely emphasized surveillance attributes and descriptive implementation (5, 16), the current study applied CFIR to conceptualize IDSR as an innovation, thereby synthesizing innovation-related characteristics that were previously reported but not analytically foregrounded as implementation barriers. These issues collectively impede the effectiveness of the system in performing the IDSR roles. To enhance IDSR implementation, it is crucial to address these challenges through system redesign, improved training programs, better integration of disease reporting across programs, expansion of the scope of the system, and increased adaptability to evolving health priorities. These improvements would boost the capability of the system to meet the current and future public health surveillance needs.

#### 4.1.2 Outer setting domain

The outer setting domain significantly impedes surveillance activities in health facilities. Key barriers found in our study include undue donor influence (External Pressure), insufficient funding (financing), negative stakeholder perceptions of the IDSR system (Local Attitudes), the absence of data-sharing frameworks, and non-compliance with international case confirmation standards (Policies & Laws). These findings corroborate previous research (24–27) that highlighted the impact of external pressure, lack of policy or inadequate policy adherence, and lack of financial support on the implementation of health system innovation. However, this study uniquely identified local attitudes as a barrier, possibly because of the use of the updated CFIR (19). This distinction underscores the importance of employing the current analytical framework to comprehensively assess the implementation challenges in healthcare settings.

#### 4.1.3 Inner setting domain

The lack of available resources for IDSR implementation, such as funding, materials and equipment, transport, and space, was identified as a crucial barrier. This resonates with previous literature, indicating the depth of under-resourcing of health facilities for IDSR-related activities (28, 29). A lack of materials and equipment, space, and transportation may arise from inadequate funding.

Lack of access to knowledge and information for the staff, such as training, feedback, supervision, and IDSR information materials, was identified as another key barrier. This finding confirms those of prior studies, highlighting the need to improve access to knowledge and information for staff (3, 30, 31). A lack of available resources may affect access to knowledge and information, which in turn limits the staff's ability to perform IDSR functions.

Challenges with health facilities' structural characteristics are among the barriers identified in this review. Deficits in Information Technology Infrastructure, including the absence of interoperability of data systems, use of old data systems/software, unstable electronic data systems, and poor network signals, result in inefficient electronic data systems that may lead to reliance on manual and paper-based systems (31–34). Other studies highlighted limitations such as health facilities' work infrastructure resulting from shortage of staff, high staff turnover, industrial action, lack of training, and lack of designated IDSR focal persons, which negatively affect the adoption, fidelity, and sustenance of IDSR as an innovation (16, 30, 35). These challenges may affect the effective implementation of IDSR, resulting in poor surveillance and case detection.

Problems with communication within the health facility were also an important barrier to the implementation of IDSR. The lack of reporting systems, inadequate feedback mechanisms, and lack of communication between surveillance and HMIS officers posed problems with the implementation process. This corroborates previous studies and calls for the need to improve health facility communication, especially among staff, to improve the implementation of IDSR. For instance, in Malawi, one study reported poor timeliness in providing monthly and weekly reports in regards to surveillance (35). Other studies have shown lack of reporting forms, inadequate reporting tools, and internet connectivity as hindrances to effective communication for enhancing implementation of IDR (28, 36).

Deficits in mission alignment, as exemplified by studies highlighting substantial reporting burden, workload, and competing tasks, have been identified as significant barriers. This observation corroborates findings from previous studies that reported that the misalignment of innovation with organizational goals adversely affects the implementation of innovation (37–39). Perceived incompatibility with organizational workflows results in resistance among implementers and a lack of leadership support, ultimately hindering adoption. Another barrier was insufficient tension for change, as staff assigned low priority to disease surveillance activities, perceiving them as redundant. This observation is consistent with the findings of Lewis et al., who noted that primary care staff deprioritized a new care model, believing they were already providing high-quality care (40). Therefore, healthcare personnel may not perceive the necessity of engaging in IDSR functions and view them as duplicative. This perception could result from the existence of multiple reporting systems for disease programs and Health Management Information Systems (HMIS). Culture among health workers in which IDSR functions as a vertical program surfaced as another barrier. Prior research has demonstrated that culture as a barrier limits trust, information sharing, staff involvement in implementation improvement, low participation in decision-making, and resistance to change (41–43). These factors may hinder the successful implementation of IDSR. It was also revealed that limitations in partnerships and connections between district health authorities and private health facilities lead to difficulties in obtaining data from private health facilities. This resonates with studies in Kenya that demonstrated that weak interdepartmental communication and lack of shared platforms can impede coordination and innovation implementation (40, 44, 45). Health authorities must employ strategies aimed at strengthening partnerships and connections with the private sector through improved communication and coordination. This may enhance data sharing for implementation, thereby creating an enabling environment to support the implementation of IDSR. Similarly, inadequate relational connections with other health-relevant sectors were another barrier to the implementation of IDSR. Previous research has also shown that weak relational and professional connections among key stakeholders may affect the implementation of IDSR activities (46–48).

#### 4.1.4 Individuals domain

We identify barriers related to innovation deliverers and facilitators. The lack of capability and motivation among the staff posed a significant challenge in the implementation of IDSR. Furthermore, the review highlighted that a lack of knowledge and skills to perform surveillance functions, such as data management and analysis, supervision, feedback, and knowledge about case definitions and guidelines, affected the ability of staff to effectively implement the IDSR system. Similar results were reported in other studies where lack of knowledge among the health workers in using surveillance technologies, data management, surveillance and reporting of notifiable diseases hindered the implementation of surveillance programs (3, 49, 50). Knowledge deficits may result in the poor detection of cases, under-reporting, and poor responses to public health emergencies.

It was also revealed that lack of motivation had an impact on IDSR performance. This has resulted in inconsistent supervision from implementation facilitators, inadequate implementation of training programs for surveillance deliverers, delays in sending data to the next higher level in time, lack of commitment to conduct community-based and active surveillance, carryout response activities, proper management of data, and low acceptance of the IDSR system. Furthermore, lack of motivation contributed to poor adherence to IDSR materials, especially guidelines and case definitions, under-recognition of priority diseases, underutilization of the laboratory for confirmation of cases, and poor data and reporting completeness. Other studies have shown similar findings, where a lack of motivation was observed among supervisors, as they were not motivated to perform supervisory roles (34, 51). This may compromise the frequency of supervision, adherence to guidelines, and implementation of enforcement measures for IDSR activities. Additionally, lack of motivation has been reported in other studies as a contributing factor to poor participation among employees in surveillance programmes (3, 16). It is evident that a lack of motivational strategies for HCWs may hinder the implementation of IDSR, as demotivated staff may not feel attracted to take part in IDSR activities such as data collection, reporting, case detection, and others.

#### 4.1.5 Implementation process

Limitations in engaging implementation deliverers, planning, and teaming were identified as barriers to the implementation of IDSR. It was found that lack of engagement with implementation deliverers, such as those in private health facilities and other one-health-related sectors (animal and environmental health sectors), was a threat to the effective implementation of IDSR. This is in line with previous studies, in which CFIR was used to understand the implementation of health information technologies. These studies found that implementation was facing challenges due to the lack of engagement of stakeholders such as various health care practitioners, in the implementation process, as they were being left out in the initiation process as well as implementation activities (52, 53). Deficits in planning were also reported in previous studies, which highlighted a lack of planning, such as the absence of a strategic plan, lack of need-based planning, and lack of a roadmap for implementation (53, 54). These factors hindered the effective implementation of innovations to support public health interventions. The review also revealed a lack of teaming, such as the absence of technical working groups, as well as rapid response teams in the implementation process, as a barrier to the implementation of IDSR. Previous research highlighted the significance of teaming where various health professionals, leaders, and designated personnel come together to facilitate IDSR implementation (55). Teaming may play a role in ensuring that relevant individuals with skills are incorporated and assigned responsibilities accordingly to enhance timely response, reporting, and case detection through IDSR.

#### 4.1.6 Outcomes

The study found that the staff's perception of the lack of usefulness (**Innovation Outcomes**) of the IDSR system was a barrier. This perception might have an impact on the staff's motivation to perform the IDSR function with fidelity, thereby affecting its implementation. This resonates with prior research, in which some health workers expressed reservations regarding the flexibility of the system to adapt to new demands and changing conditions, making them feel that the system was less useful for surveillance under various conditions (56).

### 4.2 Facilitators to the implementation of IDSR

Having good structural characteristics, including work and information technology infrastructure, has emerged as a facilitator of IDSR implementation. This is followed by access to knowledge and information, available resources, and innovation outcomes.

Other enablers included the presence of motivated staff and supervisors, the presence of policies and laws guiding IDSR implementation, tailoring strategies to overcome implementation barriers, having staff with capability in terms of good knowledge about IDSR, good quality IDSR design that is resilient and has integrated reporting systems including laboratory results, user friendliness, good planning including having a training agenda and workplans, having functional implementation teams such as rapid response teams and technical working groups, presence of good communication systems such as feedback mechanisms and electronic communication systems, having a simple system, engaging private health facilities and vertical programs, organizational culture of IDSR deliverer centeredness, actual implementation outcomes, financing from partner organizations and government, enabling local conditions, partnerships and connections with other organizations, presence of innovation adaptability, good innovation evidence base, and opportunities that come with responding to critical incidents such as COVID-29.

Many of these facilitator constructs are inversely related to barrier constructs and have been addressed previously. Constructs identified only as facilitators included evidence innovation bases, critical incidents, local conditions, tailoring strategies, and actual implementation outcomes.

#### 4.2.1 Innovation

This study demonstrated that a perceived strong evidence base for the IDSR correlates with enhanced performance in detecting and reporting public health threats, with data analysis identified as a facilitating factor. This finding aligns with previous research that examined the barriers to and facilitators of the implementation of community-based healthcare innovations (57, 58). These studies indicate that the perception of a strong evidence base supporting the efficacy of innovations serves as a facilitator for implementation (57, 58). It has been observed that such a perception motivates staff and leadership, fosters trust, and enhances the adoption of innovation (57, 58).

#### 4.2.2 Outer setting domain

The occurrence of **Critical Incidents**, such as COVID-19, posed an opportunity to strengthen the health system and provide a good implementation climate for IDSR implementation. It is evident that the emergence of COVID-19 was a wakeup call to invest and strengthen surveillance programs to prevent future outbreaks and ensure timely detection and response to emerging public health threats. This resonates well with prior studies, which highlighted that COVID-19 made countries recognize their vulnerability to public health emergencies, which forced the authorities to implement surveillance programs, allocate surveillance resources toward surveillance, and implement capacity-building programs (59–61). This suggests that conditions such as COVID-19 were facilitators of implementation, as well as strengthening surveillance programs aimed at preventing future emergencies.

Local Conditions, such as foundations from other initiatives, have emerged as facilitators of the implementation of IDSR. For instance, the existing foundation of the EWARN system was identified as a facilitator to improve the implementation of IDSR, as it facilitated case detection as well as reporting during public emergencies. Similar results were reported in a study conducted in Somalia and Syria, where the existing EWARN system played a role in facilitating COVID-19 reporting, signifying its flexibility to accommodate the integration of reportable diseases (62, 63). This enhanced timeliness, disease, flexibility, alert verification, and responding to emerging outbreaks.

It was further revealed that Partnerships and Connections are essential for enhancing surveillance initiatives. This partnership enhanced collaboration among healthcare facilities with others who provided financial and other forms of support for enhancing surveillance. This was confirmed in other studies that highlighted the significant role played by partners such as the WHO, who were actively involved in supporting surveillance programs through funding support, capacity building, and providing resources in response to public health emergencies (64, 65). The presence of policies and laws was an enabler to the implementation of the IDSR in most countries, as it enhanced the standard procedures as well as the approach to surveillance. For instance, some studies have reported the presence of technical guidelines for supporting IDSR as a tool for enhancing surveillance programs (3, 16). The guidelines provide the standards for case detection, data management, case reporting as well as response to public emergencies, which enhances surveillance (3–5, 34).

#### 4.2.3 Inner setting domain

Our study found that various issues in the inner setting domain of the CFIR facilitated IDSR implementation. Access to knowledge and information was found to be an enabler for the implementation of IDSR. Some studies have reported the availability of guidelines that are essential sources of knowledge for guiding health workers and other individuals in surveillance programs.

Other studies have highlighted the availability of essential resources as an enabler for the implementation of IDSR. These findings stress that the presence of a supportive infrastructure is essential for strengthening surveillance activities. Resources such as information technology infrastructure, mobile phones, computers, internet connectivity, and data management tools, are enablers of timely data reporting and communication within the surveillance system. Additionally, the availability of IDSR equipment, such as technical guidelines, case definition manuals, and reporting forms, is essential to ensure consistency in case identification and reporting. Another form of resource identified as an enabler for the implementation of IDSR is human resources. These findings corroborate with previous evidence, where it was found that the availability of laboratory infrastructure with diagnostic tools and integration of information and communication technology were facilitators of IDSR implementation (3, 32). Furthermore, mobile phones are available using short-message services (32). These findings are consistent with previous evidence, where it was found that the availability of laboratory infrastructure with diagnostic tools, integration of information and communication technology, availability of mobile phones, and short message services (32).

The availability of trained healthcare workers, surveillance officers, and data clerks facilitated the execution of routine surveillance tasks, such as data collection, analysis, reporting, and case detection. For instance, other studies have highlighted the significance of the availability of adequate human resources with expertise as an enabling achievement of the goals of IDSR, as this allows effectiveness in case detection as well as reporting (3, 66).

#### 4.2.4 Individuals domain

The review found that individuals' capabilities in performing various roles in IDSR were an influencing factor in its implementation. The capabilities reported include knowledge of case definitions, alert thresholds, and capabilities in using mobile gadgets. These capabilities may allow individuals to effectively detect cases, reports, and analyze data. Similar results were reported in other studies where it was noted that health workers were able to effectively use mobile technologies to report COVID-19 cases and share public health information (67, 68). The current study also found that motivation among individuals involved in IDSR is essential for enhancing its implementation. Similar findings were reported in another study which revealed that community health workers were motivated to take an active role in community health surveillance, as they were motivated through management support, training (54). Motivation among health workers is one of the key drivers that enhances support for public health responses (32, 33, 69). Individual high-level leaders further enhance the implementation of surveillance programmes. The personal commitment of leaders to supervising, monitoring, and managing IDSR programs may result in fruitful results. Previous research highlighted a significant role of high-level leaders, through support in resource planning and allocation, which enhances surveillance during public health emergencies (67, 70).

#### 4.2.5 Implementation process

Tailoring Strategies that involve identifying strategies to overcome barriers to implementation have also emerged as facilitators. Among the tailoring strategies implemented to overcome barriers to the implementation of IDSR include the use of community health volunteers to take part in case detection as well as reporting. Other studies reported similar results, where community case management teams played an important role in community case detection as well as reporting (71, 72). This strategy helps address barriers such as shortage of staff, delayed detection, and notification of cases, as community volunteers take part in community-specific surveillance initiatives. Additionally, teaming and planning were found to enhance the implementation of IDSR. Some studies have shown the collaboration and engagement of various professionals, rapid response team and well-established structure with dedicated staff as an essential strategy for enhancing IDSR implementation (68, 69, 72). This may ensure sustainability of surveillance programs in healthcare facilities.

A distinct Actual Implementation Outcome, adoption of the IDSR, for implementing public health surveillance systems in health facilities, was identified as another facilitator. It was found that a number of facilities adopted and championed the implementation of IDSR, where responsible personnel were identified and some supportive resources were allocated. This corroborates prior research that highlighted that several healthcare facilities adopted IDSR strategy for implementing public health surveillance, where guidelines and protocols were also developed to facilitate surveillance programs (49, 73). This created an environment that would allow effective implementation of surveillance initiatives. Evidence from the review has further shown that the outcomes as well as the impact of the IDSR were enabling factors in its adoption as well as implementation. The significance of the IDSR in improving case detection, enhancing timely response to outbreaks as well as playing a role in strengthening community surveillance, signifies its positive outcomes that may facilitate its adoption and implementation (4, 49, 72).

#### 4.2.6 Interactions among CFIR constructs

The interaction between access to knowledge and information and individual capability highlights the importance of sustained capacity-building approaches that extend beyond once-off training. Likewise, the observed interaction between communication and motivation underscores the critical role of feedback mechanisms as a cross-cutting determinant of engagement. Strengthening communication and feedback may therefore represent a high-leverage strategy for reinforcing motivation and improving reporting practices. Interactions between innovation design and work infrastructure further illustrate how system design choices can unintentionally increase workload and undermine usability, particularly in contexts characterized by parallel reporting systems and limited digital infrastructure. Addressing such design-infrastructure misalignments is essential to reduce reporting burden and support sustained adoption.

The CFIR construct Available Resources was identified as a key barrier, manifesting as limited funding, logistics and staffing. According to CFIR–ERIC guidance operationalised through the CFIR-ERIC matching tool (19), this barrier aligns with implementation strategies such as access new funding, develop formal implementation blueprint, and change physical equipment. In practice, these strategies could be operationalised through context-specific actions, thereby addressing the underlying mechanisms limiting implementation.

### 4.3 Strengths and limitations

To the best of our knowledge, this study represents the first application of CFIR to investigate the determinants of IDSR implementation. This research employed rigorous methodologies to collect data on both barriers and facilitators. The systematic review incorporated studies utilizing diverse data collection approaches, including qualitative, quantitative, and mixed methods designs. By incorporating data from studies conducted across Africa, this study ensured a substantial sample size, thereby enhancing the generalizability of the findings. The use of CFIR to analyze factors influencing IDSR implementation establishes a foundation for further research in this field, utilizing this framework to ensure uniform terminology in describing barriers, thus facilitating the comparison of implementation determinants across different contexts. However, interpretation of these findings should be undertaken with caution. Most included studies employed cross-sectional designs and were assessed as low to moderate quality, limiting the ability to draw causal inferences or make strong claims regarding scalability. Accordingly, the findings are best understood as reflecting recurring implementation patterns across settings rather than definitive determinants of implementation success. These patterns highlight areas that consistently warrant attention during implementation but do not establish causal pathways.

Our study had several limitations. The search was confined to studies conducted in Africa, potentially excluding relevant data from countries outside the continent that implemented national surveillance aligned with the IDSR. Furthermore, the search was limited to English-language studies, which may have reduced the sample size and introduced language bias. As this synthesis is based on the frequency of coded excerpts from predominantly cross-sectional studies, the prominence of a barrier or facilitator does not necessarily correspond to its causal weight or impact on implementation outcomes. Despite these limitations, the findings remain valuable for applications in Africa and similar settings to identify strategies for overcoming barriers and enhancing facilitators to improve IDSR implementation.

## 5 Conclusion

The review found that IDSR implementation encounters complex challenges and opportunities within healthcare facilities. The inner setting of health facilities has emerged as a critical factor, both as barriers and enablers. Crucial barriers include limited available resources, inadequate access to knowledge and information, and constraints on structural characteristics. Individual factors, particularly motivation and capability, pose significant challenges. However, this review suggests that the same inner setting elements can act as enablers when properly addressed. Structural characteristics, improved access to knowledge and adequate resources can facilitate IDSR implementation. Additionally, positive innovation outcomes serve as important enablers.

These findings highlight the multifaceted nature of IDSR implementation, emphasizing the need for comprehensive strategies that address both organizational and individual factors. Future efforts should focus on strengthening health facility infrastructure, enhancing knowledge dissemination, boosting individual motivation and capability to overcome barriers, and leveraging existing enablers for successful IDSR implementation. The CFIR-ERIC matching tool can be applied to identify theory-based tailored strategies, depending on the context.

## Data Availability

The raw data supporting the conclusions of this article will be made available by the authors, without undue reservation.
