# Supplementary material for: Barriers and facilitators to the implementation of integrated disease surveillance and response (IDSR) in Africa: a systematic review using the consolidated framework for implementation research (CFIR)
Source: Front Public Health. 2026 Feb 20;14:1758944. doi: 10.3389/fpubh.2026.1758944 (PMC12963351; doi:10.3389/fpubh.2026.1758944)
Supplement: Supplementary file 1 [file Data_Sheet_1.docx]

**Supplementary file 1: Search strategy for various electronic databases**

1. **Scopus search strategy**

| Number |  | Results |
| --- | --- | --- |
| #1 | TITLE-ABS-KEY ( ‘africa’ OR angola OR benin OR botswana OR "BURKINA FASO" OR "CABO VERDE" OR cameroon OR "CENTRAL AFRICAN REPUBLIC" OR chad OR congo OR "COTE D'IVOIRE" OR "DEMOCRATIC REPUBLIC OF THE CONGO" OR "DJIBOUTI" OR "EQUATORIAL GUINEA" OR "ERITREA" OR "ESWATINI" OR "ETHIOPIA" OR "GABON" OR "GAMBIA" OR "GHANA" OR "GUINEA" OR "GUINEA-BISSAU" OR "KENYA" OR "LESOTHO" OR "LIBERIA" OR "MALAWI" OR "MALI" OR mauritania OR mozambique OR namibia OR niger OR nigeria OR rwanda OR "SAO TOME AND PRINCIPE" OR senegal OR "SIERRA LEONE" OR somalia OR "SOUTH AFRICA" OR "SOUTH SUDAN" OR sudan OR tanzania OR togo OR uganda OR zambia OR zimbabwe OR algeria OR egypt OR libya OR morocco OR tunisia ) | 1,509,373 |
| #2 | TITLE-ABS-KEY ( "IDSR " OR "integrated disease surveillance and response" OR "surveillance system" OR "public health surveillance" OR "public health surveillance system" OR "IDS" OR "Integrated disease surveillance" ) | 201,123 |
| #3 | TITLE-ABS-KEY ("Interview" OR "survey* OR questionnaire" OR " multi stage cluster sampling " OR " cross sectional survey " OR " focus group discussion " OR " observation study " OR survey) | [4,012,467](https://www.scopus.com/search/history/results.uri?origin=searchhistory&shid=9) |
| #5 | TITLE-ABS-KEY ( "rapid assessment" OR "Experiences" OR "Gaps" OR "Successes" OR "IDSR performance" OR "indicators" OR "knowledge acquisition" OR "knowledge retention" OR "level of confidence" OR "views" OR "challenges" OR "perception" OR "benefits" OR "solutions" OR "perspective" OR "factor*" ) | [27,955,000](https://www.scopus.com/search/history/results.uri?origin=searchhistory&shid=5) |
| #6 | #1 AND #2 | [6,166](https://www.scopus.com/search/history/results.uri?origin=searchhistory&shid=6) |
| #7 | #3 OR #5 | [29,949,161](https://www.scopus.com/search/history/results.uri?origin=searchhistory&shid=10) |
| #8 | #6 AND #7 | 4089 |

1. **Web of Science search strategy**

| Number |  | Results |
| --- | --- | --- |
| #1 | ‘africa’ OR angola OR benin OR botswana OR "BURKINA FASO" OR "CABO VERDE" OR cameroon OR "CENTRAL AFRICAN REPUBLIC" OR chad OR congo OR "COTE D'IVOIRE" OR "DEMOCRATIC REPUBLIC OF THE CONGO" OR "DJIBOUTI" OR "EQUATORIAL GUINEA" OR "ERITREA" OR "ESWATINI" OR "ETHIOPIA" OR "GABON" OR "GAMBIA" OR "GHANA" OR "GUINEA" OR "GUINEA-BISSAU" OR "KENYA" OR "LESOTHO" OR "LIBERIA" OR "MALAWI" OR "MALI" OR mauritania OR mozambique OR namibia OR niger OR nigeria OR rwanda OR "SAO TOME AND PRINCIPE" OR senegal OR "SIERRA LEONE" OR somalia OR "SOUTH AFRICA" OR "SOUTH SUDAN" OR sudan OR tanzania OR togo OR uganda OR zambia OR zimbabwe OR algeria OR egypt OR libya OR morocco OR tunisia (Topic) | 1,140,003 |
| #2 | TS=("IDSR " OR "integrated disease surveillance and response" OR "surveillance system" OR "public health surveillance" OR "public health surveillance system" OR "IDS" OR "Integrated disease surveillance" ) | 48,736 |
| #3 | TS=("Interview" OR "survey* OR questionnaire" OR " multi stage cluster sampling " OR " cross sectional survey " OR " focus group discussion " OR " observation study " OR survey ) | [2,216,881](https://wos1.research4life.org/wos/alldb/summary/a31435b4-6e32-422d-99b8-94ece3656378-0107ed91dd/relevance/1) |
| #4 | TS=("rapid assessment" OR "Experiences" OR "Gaps" OR "Successes" OR "IDSR performance" OR "indicators" OR "knowledge acquisition" OR "knowledge retention" OR "level of confidence" OR "views" OR "challenges" OR "perception" OR "benefits" OR "solutions" OR "perspective" OR "factor*" ) | [14,355,957](https://wos1.research4life.org/wos/alldb/summary/714c462d-ea21-4ee9-88cb-3cc82964b3cb-0107edb8e8/relevance/1) |
| #5 | #1 AND #2 | [3,606](https://wos1.research4life.org/wos/alldb/summary/f9393e25-6b77-4a9c-8178-a4c0a0d0980b-0107ede763/relevance/1) |
| #6 | #3 OR #4 | [15,495,868](https://wos1.research4life.org/wos/alldb/summary/05998c86-a7cb-412b-a0fc-3e37d3f6d1d8-0107ee00a2/relevance/1) |
| #7 | #5 AND #6 | 2,387 |

1. **CINAHL Search strategy**

| Number |  | Results |
| --- | --- | --- |
| S1 | TI ( ‘africa’ OR angola OR benin OR botswana OR "BURKINA FASO" OR "CABO VERDE" OR cameroon OR "CENTRAL AFRICAN REPUBLIC" OR chad OR congo OR "COTE D'IVOIRE" OR "DEMOCRATIC REPUBLIC OF THE CONGO" OR "DJIBOUTI" OR "EQUATORIAL GUINEA" OR "ERITREA" OR "ESWATINI" OR "ETHIOPIA" OR "GABON" OR "GAMBIA" OR "GHANA" OR "GUINEA" OR "GUINEA-BISSAU" OR "KENYA" OR "LESOTHO" OR "LIBERIA" OR "MALAWI" OR "MALI" OR mauritania OR mozambique OR namibia OR niger OR nigeria OR rwanda OR "SAO TOME AND PRINCIPE" OR Senegal OR "SIERRA LEONE" OR somalia OR "SOUTH AFRICA" OR "SOUTH SUDAN" OR sudan OR tanzania OR togo OR uganda OR zambia OR zimbabwe OR algeria OR egypt OR libya OR morocco OR tunisia ) OR AB ( ‘africa’ OR angola OR benin OR botswana OR "BURKINA FASO" OR "CABO VERDE" OR cameroon OR "CENTRAL AFRICAN REPUBLIC" OR chad OR congo OR "COTE D'IVOIRE" OR "DEMOCRATIC REPUBLIC OF THE CONGO" OR "DJIBOUTI" OR "EQUATORIAL GUINEA" OR "ERITREA" OR "ESWATINI" OR "ETHIOPIA" OR "GABON" OR "GAMBIA" OR "GHANA" OR "GUINEA" OR "GUINEA-BISSAU" OR "KENYA" OR "LESOTHO" OR "LIBERIA" OR "MALAWI" OR "MALI" OR mauritania OR mozambique OR namibia OR niger OR nigeria OR rwanda OR "SAO TOME AND PRINCIPE" OR Senegal OR "SIERRA LEONE" OR somalia OR "SOUTH AFRICA" OR "SOUTH SUDAN" OR sudan OR tanzania OR togo OR uganda OR zambia OR zimbabwe OR algeria OR egypt OR libya OR morocco OR tunisia ) | 98,913 |
| S2 | TI ( "IDSR " OR "integrated disease surveillance and response" OR "surveillance system" OR "public health surveillance" OR "public health surveillance system" OR "IDS" OR "Integrated disease surveillance" ) OR AB ( "IDSR " OR "integrated disease surveillance and response" OR "surveillance system" OR "public health surveillance" OR "public health surveillance system" OR "IDS" OR "Integrated disease surveillance" ) | 10,618 |
| S3 | TI ( "Interview" OR "survey* OR questionnaire" OR " multi stage cluster sampling " OR " cross sectional survey " OR " focus group discussion " OR " observation study " OR survey ) OR AB ( "Interview" OR "survey* OR questionnaire" OR " multi stage cluster sampling " OR " cross sectional survey " OR " focus group discussion " OR " observation study " OR survey ) | 399,553 |
| S4 | TI ( =("rapid assessment" OR "Experiences" OR "Gaps" OR "Successes" OR "IDSR performance" OR "indicators" OR "knowledge acquisition" OR "knowledge retention" OR "level of confidence" OR "views" OR "challenges" OR "perception" OR "benefits" OR "solutions" OR "perspective" OR "factor*" ) OR AB ( "rapid assessment" OR "Experiences" OR "Gaps" OR "Successes" OR "IDSR performance" OR "indicators" OR "knowledge acquisition" OR "knowledge retention" OR "level of confidence" OR "views" OR "challenges" OR "perception" OR "benefits" OR "solutions" OR "perspective" OR "factor*" ) | 1,436,953 |
| S5 | S1 AND S2 | [708](https://wos1.research4life.org/wos/alldb/summary/f9393e25-6b77-4a9c-8178-a4c0a0d0980b-0107ede763/relevance/1) |
| S6 | S3 OR S4 | 1,661,171 |
| S7 | (S1 AND S2) AND (S3 OR S4) | 376 |

**Supplementary file 2: Quality assessment of studies that used quantitative methods.**

1. **Quantitative studies**

| **Criteria** | Rumunu et al. (2022), South Sudan (32) | Riolexus et al. (2022); Uganda(24) | Ibrahim et al. (2020); Nigeria(35) | Stolka et al. (2018); DRC(36) | Twene et al. (2024); Ghana(38) | Nagbe et al. (2019); Liberia(39) | Benson et al. (2016); South Africa(40) | Mwatondo et al. (2016); Kenya(42) | Mremi et al. (2022); Tanzania(49) | Yusuf et al. (2023); Ethiopia(50) | Lakew et at. (2017); Ethiopia(51) | Martin et al. (2020); Sierra Leone(52) | Lafond et al. (2014); Nigeria(59) | Toda et al. (2018); Kenya(60) | Brown (2017); South Africa(63) | Kareko et al (2019); Kenya(23) |
| --- | --- | --- | --- | --- | --- | --- | --- | --- | --- | --- | --- | --- | --- | --- | --- | --- |
| Does the researcher identify what is known and not known about the problem? | Yes | Yes | Yes | Yes | No | No | Yes | Yes | Yes | Yes | Yes | Yes | Yes | Yes | No | Yes |
| Does the researcher identify how the study will address any gaps in knowledge? | Yes | Yes | Yes | Yes | No | No | Yes | Yes | Yes | Yes | Yes | Yes | Yes | Yes | No | Yes |
| Was the purpose of the study clearly presented? | Yes | Yes | Yes | Yes | No | No | Yes | Yes | Yes | Yes | Yes | Yes | Yes | Yes | No | Yes |
| Was the literature review current (most sources within the past five years or a seminal study)? | Yes | Yes | Yes | Yes | Yes | Yes | Yes | Yes | Yes | Yes | Yes | Yes | Yes | Yes | No | Yes |
| Was sample size sufficient based on study design and rationale? | No | Yes | No | No | Yes | Yes | No | Yes | No | Yes | No | No | No | No | No | No |
| Are data collection methods described clearly? | Yes | Yes | Yes | Yes | Yes | Yes | Yes | Yes | Yes | Yes | Yes | Yes | Yes | Yes | No | Yes |
| Were the instruments reliable (Cronbach’s a [alpha] > 0.70)? | No | No | No | No | No | No | Yes | No | No | No | No | No | No | No | No | No |
| Was instrument validity discussed? | No | No | No | Yes | Yes | No | Yes | No | No | Yes | No | No | Yes | No | No | No |
| If surveys or questionnaires were used, was the response rate > 25%? | Yes | Yes | Yes | Yes | Yes | N/A | Yes | Yes | Yes | Yes | Yes | Yes | Yes | Yes | No | Yes |
| Were the results presented clearly? | Yes | Yes | Yes | Yes | Yes | Yes | Yes | Yes | Yes | Yes | Yes | Yes | Yes | Yes | No | Yes |
| If tables were presented, was the narrative consistent with the table content? | Yes | Yes | Yes | Yes | Yes | Yes | Yes | Yes | Yes | Yes | Yes | Yes | Yes | Yes | No | Yes |
| Were study limitations identified and addressed? | Yes | No | Yes | Yes | No | No | Yes | Yes | Yes | Yes | Yes | Yes | Yes | Yes | No | Yes |
| Were conclusions based on results? | Yes | Yes | Yes | Yes | Yes | Yes | Yes | Yes | Yes | Yes | Yes | Yes | Yes | Yes | No | Yes |
| Overall evidence strength level | III | III | III | III | III | III | III | III | III | III | III | III | III | III | III | III |
| Overall evidence quality | C | A | C | C | B | B | C | B | C | B | C | C | C | C | C | C |

1. **Qualitative studies**

| **Criteria** | Meierkord et al. (2024); Côte d'Ivoire, Ecuador, Madagascar, Namibia, and the Kingdom of Saudi Arabia(31) | Kambalame et al. (2024); Malawi(33) | Nyenswah et al. (2023); Liberia(25) | Adokiya et al. (2015); Ghana(37) | Nakiire et al. (2019); Uganda(47) | Mandyata et al. (2017); Zambia(56) |
| --- | --- | --- | --- | --- | --- | --- |
| Was there a clearly identifiable and articulated: Purpose? | yes | Yes | Yes | Yes | Yes | yes |
| Was there a clearly identifiable and articulated: Research question? | yes | Yes | Yes | Yes | Yes | yes |
| Was there a clearly identifiable and articulated: Justification for design and/or theoretical framework used? | No | Yes | Yes | Yes | Yes | Yes |
| Do participants have knowledge of the subject the researchers are trying to explore? | yes | Yes | Yes | Yes | Yes | yes |
| Were characteristics of study participants described? | yes | Yes | Yes | Yes | Yes | yes |
| Was a verification process used in every step of data analysis (e.g., triangulation, response validation, independent double check, member checking)? (Credibility) | yes | Yes | Yes | No | No | Yes |
| Does the researcher provide sufficient documentation of their thinking, decisions, and methods related to the study allowing the reader to follow their decision-making (e.g., how themes and categories were formulated)? (Confirmability) | yes | Yes | Yes | Yes | yes | yes |
| Does the researcher provide an accurate and rich description of findings by providing the information necessary to evaluate the analysis of data? (Fittingness) | yes | Yes | Yes | yes | yes | Yes |
| Does the researcher acknowledge and/or address their own role and potential influence during data collection? | No | No | Yes | Yes | No | No |
| Was sampling adequate, as evidenced by achieving data saturation? | Yes | Yes | Yes | Yes | Yes | Yes |
| Does the researcher provide illustrations from the data? | Yes | Yes | Yes | Yes | Yes | Yes |
| ·       If yes, do the provided illustrations support conclusions? | Yes | Yes | Yes | Yes | Yes | Yes |
| Is there congruency between the findings and the data? | Yes | Yes | Yes | Yes | Yes | Yes |
| Is there congruency between the research methodology and: The research question(s) | Yes | Yes | Yes | Yes | Yes | Yes |
| Is there congruency between the research methodology and: The methods to collect data | Yes | Yes | Yes | Yes | Yes | Yes |
| Overall evidence strength level | III | III | III | III | III | III |
| Overall evidence quality | B | B | A | B | B | B |

1. **Mixed methods studies**

| **Criteria** | Zalwango et al. (2024); Uganda(34) | Siya et al. (2021); Uganda(41) | Jinadu et al. (2018); Nigeria(43) | Beebeejaun et al. (2021); Nigeria(44) | Chimsimbe et al. (2022); Zimbabwe(45) | Ssendagire et al. (2023); Somalia(46) | Ndegwa et al. (2023); Kenya(48) | Kallay et al. (2024); DRC (53) | Adokiya et al. (2015); Ghana(54) | Saleh et al. (2021); Zanzibar(55) | Wu et al. (2018); Malawi(57) | Issah et al. (2015); Ghana(58) | Ng’etich et al. (2021); Kenya(61) | Masiira et al. (2019); Uganda(62) | Ng’etich et al. (2020); Kenya(64) | Idenyi et al. (2021); Nigeria(65) |
| --- | --- | --- | --- | --- | --- | --- | --- | --- | --- | --- | --- | --- | --- | --- | --- | --- |
| Was the mixed-methods research design relevant to address both quaNtitative and quaLitative research questions (or objectives)? | Yes | Yes | Yes | Yes | Yes | Yes | Yes | Yes | Yes | Yes | Yes | Yes | Yes | Yes | Yes | Yes |
| Was the research design relevant to address the quaNtitative and the quaLitative aspects of the mixed-methods question (or objective)? | Yes | Yes | Yes | Yes | Yes | Yes | Yes | Yes | Yes | Yes | Yes | Yes | Yes | Yes | Yes | Yes |
| Quanti Evidence Level | III | III | III | III | III | III | III | III | III | III | III | III | III | III | III | III |
| Quanti Evidence Quality | C | C | B | C | B | C | C | C | C | C | B | B | C | C | C | C |
| Quali Evidence level | III | III | III | III | III | III | III | III | III | III | III | III | III | III | III | III |
| Quali Evidence quality | B | C | B | B | C | B | C | B | B | B | B | B | B | B | B | C |
| Overall evidence strength level | III | III | III | III | III | III | III | III | III | III | III | III | III | III | III | III |
| Overall evidence quality | C | C | B | C | C | C | C | C | C | C | B | B | C | B | C | C |
